# Impaired SREBP1-mediated regulation of lipid metabolism promotes inflammation in chronic endometritis

**DOI:** 10.3389/fimmu.2025.1547949

**Published:** 2025-06-05

**Authors:** Shigeru Matsuda, Yoshimitsu Kuwabara, Yoshitaka Taketomi, Yuki Nagasaki, Yosuke Sugita, Shunji Suzuki, Ichiro Manabe, Makoto Murakami, Yumiko Oishi

**Affiliations:** ^1^ Department of Obstetrics and Gynecology, Nippon Medical School, Tokyo, Japan; ^2^ Laboratory of Microenvironmental and Metabolic Health Sciences, Center for Disease Biology and Integrative Medicine, Graduate School of Medicine, The University of Tokyo, Tokyo, Japan; ^3^ Department of Systems Medicine, Graduate School of Medicine, Chiba University, Chiba, Japan; ^4^ Department of Medical Biochemistry, Graduate School of Medical and Dental Sciences, Institute of Science Tokyo, Tokyo, Japan

**Keywords:** PUFA, EPA, chronic endometritis, infertility treatment, reccurrent pregnancy loss

## Abstract

Chronic endometritis (CE) is an inflammatory disease of the uterus that is associated with infertility and poor reproductive outcomes. Although most cases of CE are attributed to bacterial infections, antibiotic treatment is sometimes ineffective, and the mechanisms underlying the development and persistence of inflammation in CE are poorly understood. In the present study, we established a novel mouse model of CE that causes fetal death without affecting implantation and demonstrated that dysregulation of lipid metabolism contributes to its pathology. A deficiency in SREBP1, a key regulator of lipid metabolism, prolonged endometrial inflammation with CD138^+^ plasma cell accumulation and induced miscarriage in LPS-induced endometritis, thereby mimicking CE. Lipidomic analyses showed that *Srebf1* deficiency significantly reduced phospholipids containing eicosapentaenoic acid (EPA) within uterine tissue. Dietary supplementation of EPA increased endometrial levels of EPA-containing phospholipids and ameliorated inflammation and miscarriage in *Srebf1* ^-/-^ CE mice. These results suggest that dysregulation of lipid metabolism, particularly reductions in polyunsaturated fatty acids in endometrial phospholipids, promotes inflammation and miscarriage in CE. Importantly, EPA-containing phospholipids were also decreased in endometrial tissue from human CE patients. Thus, dysregulated lipid metabolism appears to play a pivotal role in the development of CE and provides novel therapeutic targets.

## Introduction

Chronic endometritis (CE) is a prolonged mild inflammation of the endometrium characterized by the presence of edema; increased numbers of stromal cells, particularly infiltrated plasma cells; and dissociation between maturation of epithelial cells and stromal fibroblasts (1, 2). Whereas in the normal endometrium, a small number of immune cells are located at exclusively at the basal layer, in CE a large population of plasma cells are present in both the basal layer and glandular epithelium. Although in most cases patients with CE are asymptomatic or have only mild symptoms (3), it has attracted attention due to its association with unexplained infertility, recurrent miscarriage and poor outcomes in assisted reproductive technology. For instance, rates of both pregnancy and live birth are reportedly lower in the patients with persistent endometrial inflammation than in those without inflammation (pregnancy rate 65% vs 33%, live birth rate 61% vs 13%) (4). Chronic endometritis (CE) has been reported in 30–57% of infertile women with recurrent implantation failure (RIF), indicating a high prevalence among individuals with impaired endometrial receptivity (4). Furthermore, accumulating epidemiologic evidence suggests that CE plays a role in the pathogenesis of recurrent pregnancy loss (RPL), with a significantly higher prevalence of CE reported in women with unexplained RPL, ranging from 10% to over 50%, compared with fertile controls (5). Several pathophysiological mechanisms have been proposed to explain the association between CE and RPL, including impaired decidualization, disruption of immune tolerance, and abnormalities in the endometrial microbiota (6, 7). Notably, the abnormal activation of immune cells and excessive production of pro-inflammatory cytokines may compromise the immune tolerance essential for pregnancy maintenance, thereby impairing post-implantation embryonic development and increasing the risk of miscarriage (5) (8). However, the mechanisms underlying persistent inflammation in the endometrium are poorly understood, given the tissue’s continuous remodeling through the cell proliferation and differentiation and shedding (i.e., menstruation) during each menstrual cycle. The mechanism by which persistent inflammation impairs implantation and pregnancy is also largely unknown.

Although CE can be associated with the presence of intrauterine devices as well as structural pathology within the endometrial cavity, including submucous myomas and polyps (2), most cases of CE are attributed, at least initially, to bacterial infections, which makes broad-spectrum antibiotics, such as doxycycline, the first line of treatment (9). However, inflammation persists in approximately 25% of CE patients, even after repeated administration of up to three different types of antibiotics (4). Moreover, recent studies have shown that the uterus is not a sterile cavity and may harbor a unique microbiota (10). As such, the presence of bacteria is not necessarily the cause or the persistent mechanism of this disease. There is also growing concern over the emergence of antibiotic-resistant bacteria due to the prolonged use of broad-spectrum antibacterial drugs (11). Consequently, there is an urgent need to elucidate the mechanisms that lead to the persistent unresolved inflammation characteristic of CE and to develop novel treatment strategies to treat it.

Eicosapentaenoic acid (EPA) is an omega-3 polyunsaturated fatty acid (PUFA) found in fish oils and has potent anti-inflammatory properties (12). Low serum EPA/arachidonic acid levels are reportedly associated with an increased risk for cardiovascular disease (13), and oral EPA has been shown to reduce cardiovascular risk and to lower both C-reactive protein (CRP) and low-density lipoprotein (LDL) levels (14, 15). The mechanism underlying the anti-inflammatory action of EPA appears to be multifaceted and is not fully understood, though EPA metabolites, including specialized lipid mediators such as resolvins and protectins, have been shown to have anti-inflammatory potential (16, 17). Recent studies also suggest that EPA may increase the fluidity of cell membranes by being incorporated into phospholipids, thereby altering cellular function and making cells less susceptible to inflammation, perhaps through modulation of cellular signaling events and membrane protein function (18, 19). The composition of fatty acids in membrane phospholipids varies among organs, with PUFAs being particularly abundant in reproductive organs (20, 21). Interestingly, adherence to a “Mediterranean diet,” which is rich in ω-3 PUFAs such as EPA and docosahexaenoic acid (DHA), is associated with a higher likelihood of achieving clinical pregnancy and live birth in women over 35 years of age at the first *in vitro* fertilization (22).

Sterol regulatory element-binding protein 1 (SREBP1) is a key transcription factor that regulates lipid metabolism. SREBP1 is also involved in the regulation of inflammatory responses of immune cells, particularly macrophages and T lymphocytes (23–25). We previously showed that SREBP1 drives fatty acid elongation and desaturation, promoting synthesis of PUFAs in macrophages (24). Moreover, *Srebf1^-/-^* macrophages exhibited enhanced and prolonged inflammatory responses following pro-inflammatory TLR4 stimulation (24, 26), while *Srebf1^-/-^* mice exhibited exaggerated and prolonged inflammation in a sepsis model. The enhanced inflammatory responses in *Srebf1^-/-^* mice were ameliorated by supplementation of exogenous EPA (24), suggesting that SREBP1 controls inflammatory responses in part by regulating fatty acid metabolism.

In the present study, we found that a lack of *Srebf1* exacerbates inflammation and reduces live births in a novel mouse model of CE. *Srebf1* deficiency strongly affected fatty acid species, including PUFAs, and EPA supplementation ameliorated inflammation and miscarriage in *Srebf1^-/-^* mice. In addition, lipidomics analysis revealed that levels of EPA-containing phospholipids were decreased in endometrial tissues from CE patients, suggesting that a decrease in the EPA content of the endometrium may contribute to human CE pathology.

## Results

### Establishment of a mouse model of LPS-induced endometritis

**Figure 1:**
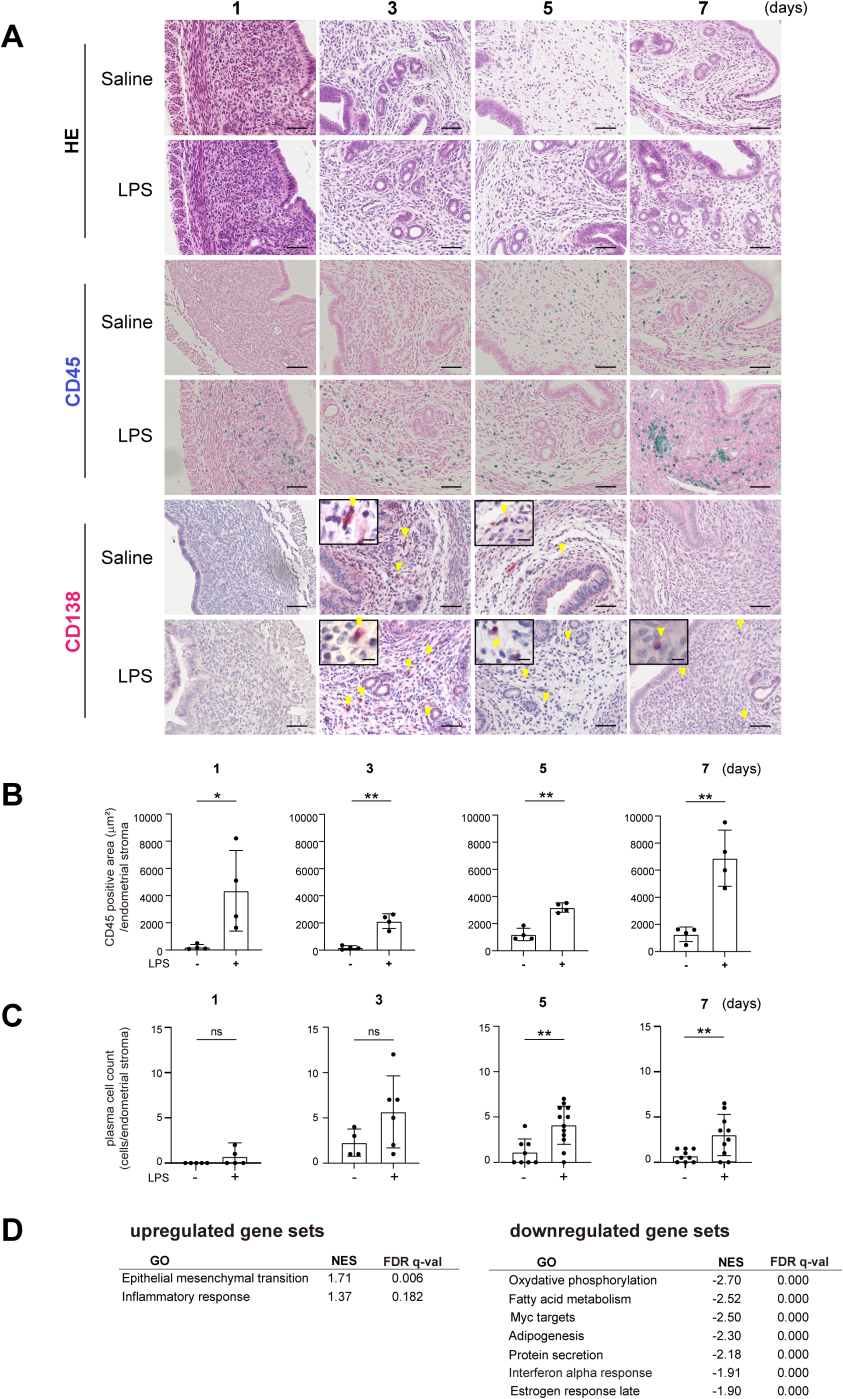
Establishment of a mouse chronic endometritis model. **(A)** Histological images of uteri from C57BL/6 (WT) mice collected on days 1, 3, 5 and 7 after intrauterine administration of saline or LPS (representative images). Mice were fed with fish meal-free chow diet for 7 days prior to saline or LPS administration. Shown are representative H-E-stained sections and immunostained for CD45 (blue) and CD138 (red). Scale bars: 50 µm. Yellow arrowheads on CD138-immunostained sections show plasma cells; magnified images are in the upper left. Scale bars: 10 µm. n=6 mice in each condition. **(B, C)** CD45-positive areas **(B)** and the plasma cell counts **(C)** in the endometrial stromal area of uteri collected on days 1, 3, 5 and 7 after intrauterine LPS administration. Data are presented as means ± SEM. Significance was determined using Student’s *t*-tests. *p<0.05, **p < 0.01; ns, not significant. n = 4–11 mice /experimental condition. Two sections were made from each side of the bicornuate uterus per mouse. Measured were the CD45-positive areas and the CD138-positive cell counts in the subepithelial stromal region of the endometrium surrounding the uterine lumen in each specimen. **(D)** MSigDB Hallmark gene sets enriched in tissues treated with LPS (FDR < 0.20) as compared to saline treatment. NES, normalized enrichment score.

With the goal of recapitulating the characteristics of human CE pathology, we first established a mouse model of uterine inflammation. To induce uterine inflammation, we administered lipopolysaccharide (LPS, 10 μg/unilateral uterine horn) or saline into the uterus through laparotomy in 8-week-old female mice. Endometrial tissue was then collected and subjected to the histological examination, including hematoxylin-eosin (H-E) staining and immunohistochemical staining for CD45, a pan-leukocyte marker, and CD138, a plasma cell marker. The presence of CD138^+^ plasma cells in the subepithelial stromal region is one of the diagnostic criteria for CE in humans (1, 27). No CD138^+^ cells were observed within uninflamed tissues (**Supplementary Figure 1**). One day after intrauterine LPS administration, CD45^+^ mononuclear cells had accumulated in the subepithelial stromal region of the endometrium, which supports the idea that LPS induced endometrial inflammation (**Figures 1A, B**). Administration of PBS also modestly increased the number of CD45^+^ cells, particularly at later time points, likely due to the surgical intervention. Notably, PBS administration did not trigger an acute accumulation of CD45^+^ cells on day 1. By contrast, a clear increase in CD45^+^ cells was observed in the LPS-treated mice across all the observed time points. In the LPS-treated mice, the number of CD138^+^ plasma cells, located primarily in the stromal region of the endometrium, peaked on day 3 and gradually decreased thereafter (**Figures 1A, C**). These results suggest that LPS injection induces an acute inflammatory response in the endometrium, followed by the accumulation of plasma cells on day 3. Although the plasma cell count tended to decrease, it remained elevated on days 5 and 7, indicating persistent inflammation.

To further investigate the effect of LPS injection, we performed bulk RNA sequencing (RNA-seq) of whole uterine tissues on day 7. Gene set enrichment analysis (GSEA) of the Molecular Signatures Database (MSigDB) hallmark gene sets using GSEApy (28–30) showed significant downregulation of gene sets related to fatty acid metabolism in the LPS-treated group as compared to the control group (**Figure 1D**), suggesting lipid metabolism was modulated in the endometritis tissues. The analysis also showed the upregulation of gene ontology (GO) terms that included “epithelial-mesenchymal transition” and “inflammatory response,” which suggests increased inflammation and remodeling.

### *Srebf1* deficiency exaggerates and sustains LPS-induced endometritis

**Figure 2:**
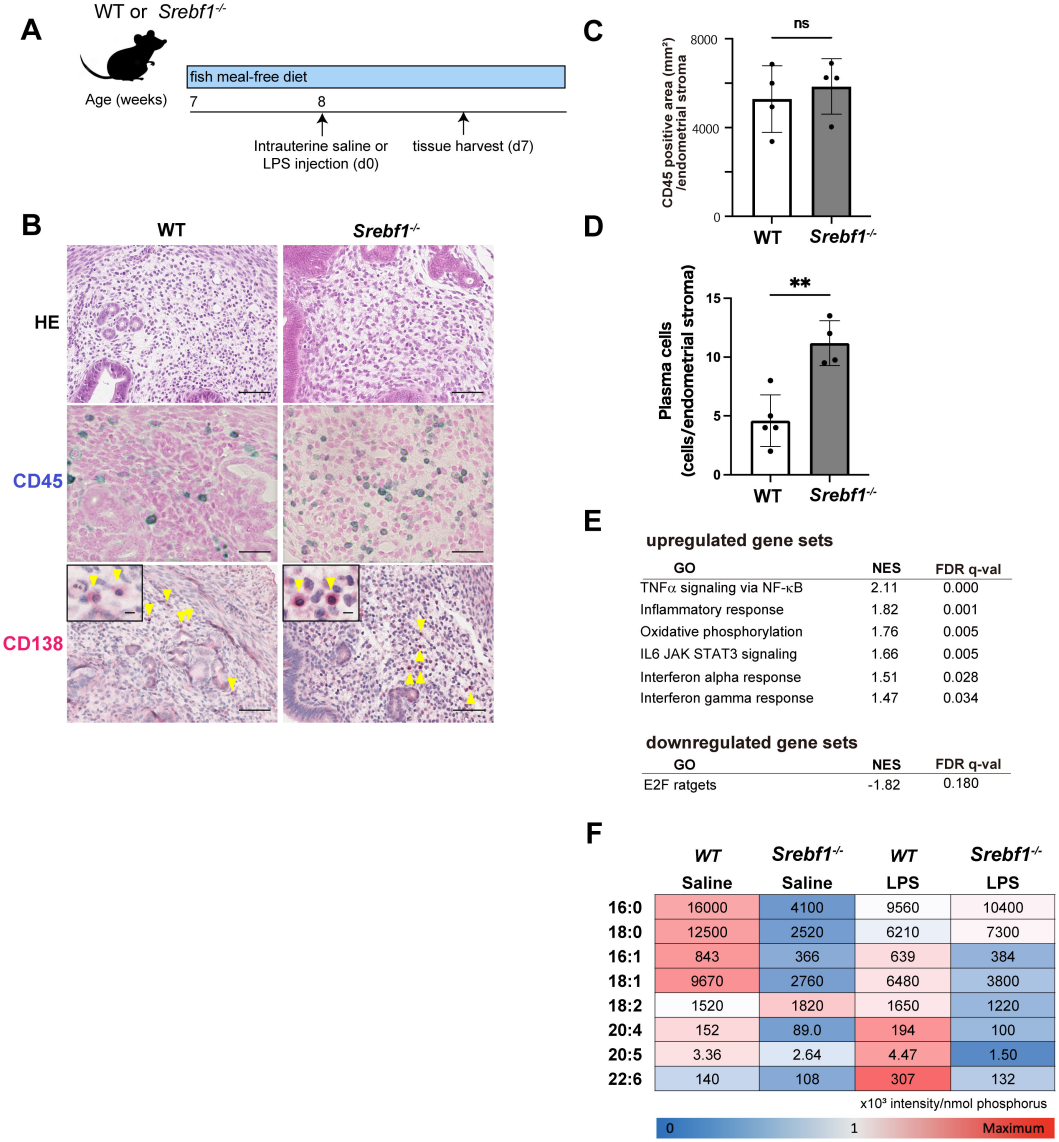
Systemic deletion of SREBP1 exacerbates and prolongs endometrial inflammation. **(A)** Schematic diagram of the LPS or saline administration used to analyze chronic endometritis in mice. **(B)** H-E-staining and immunostaining for CD45 (blue) and CD138 (red) in uteri collected from WT and *Srebf1^-/-^* mice 7 days after saline or LPS injection (scale bars: 50 µm). Yellow arrowheads on images immunostained for CD138 show plasma cells; magnified images are in the upper left (scale bars: 10 µm). **(C, D)** CD45-positive areas **(C)** and plasma cell counts **(D)** in the endometrial stromal area of uteri collected from WT and *Srebf1^-/-^* mice on day 7 after LPS administration. Mice were fed a fish meal-free diet. Data are presented as means ± SEM, and significance was determined using Student’s *t*-tests. **p<0.01, ns; not significant. n = 4–5 for each experimental condition. Two sections were made from each side of the bicornuate uterus per mouse. Measured were CD45-positive areas and CD138-positive cell counts in the subepithelial stromal region of the endometrium surrounding the uterine lumen in each specimen. **(E)** MSigDB Hallmark gene sets differentially expressed in uterine tissue from female WT and *Srebf1^-/-^* mice on day 7 after intrauterine administration of LPS (FDR < 0.20). NES, normalized enrichment score. **(F)** Lipidomics analysis showing fatty acid levels. Uterine tissues were collected from female WT and *Srebf1^-/-^* mice on day 7 after intrauterine administration of NS or LPS (n=6 mice/experimental condition). The mean and standard deviation of the signal intensities for each fatty acid species were used to calculate the z-score for each fatty acid species. Minimum (blue) and maximum (red) z-scores were determined for each fatty acid species, and a heat map was created based on the z-score. The numbers shown on the heat map represent the average signal intensities for each fatty acid species.

The finding of altered expression of genes related to lipid metabolism in the inflamed endometrium prompted us to investigate the involvement of SREBP1, a key transcription factor controlling the *de novo* biosynthesis of fatty acids including PUFAs, using *Srebf1*-deficient mice (*Srebf1^-/-^*). Because regular mouse chow contains a high level of PUFAs (50% of the 4.6 g of fat per 100 g of chow are PUFAs, mainly derived from fish meal; the rest are monounsaturated (29%) and saturated (21%) fatty acids (31)), we changed it to fish meal-free chow 7 days prior to LPS administration to minimize the effect of exogenous ω3 PUFAs (**Figure 2A**), which would allow a more precise evaluation of SREBP-1-dependent endogenous ω3 PUFA metabolism. Eight-week-old female *Srebf1^-/-^* and WT mice were administered intrauterine LPS or saline, and uterine tissues were collected 7 days post-injection (**Figure 2A**). Whereas tissues from both WT and *Srebf1* ^-/-^ mice showed similar levels of CD45^+^ mononuclear cell accumulation (**Figures 2B, C**), CD138^+^ plasma cell counts were significantly higher in the subepithelial stromal regions in *Srebf1^-/-^* mice (**Figures 2B, D**).

To characterize the effect of *Srebf1* deficiency on the endometrial inflammation, we performed bulk RNA-seq of WT and *Srebpf1^-/-^* uterine tissues on day 7 after LPS administration. GSEA of MSigDB hallmark gene sets showed that inflammatory response-related gene sets, including tumor necrosis factor-alpha (TNFα) signaling via NF-κB, inflammatory responses, interleukin (IL)-6/Janus kinase/signal transducers and activators of transcription 3 (JAK/STAT3) signaling, and interferon responses, were upregulated in uterine tissue from *Srebf1^-/-^* mice (**Figure 2E**). These transcriptome changes and the persistent accumulation of CD138^+^ plasma cells on day 7 (**Figure 2B**) suggests sustained inflammation in *Srebp1^-/-^* mice, which mimics the pathological findings in human CE patients.

### Fatty acid metabolism is dysregulated in the endometrium of *Srebf1^-/-^* mice

Given our earlier findings that *Srebf1* deletion decreases PUFA levels in skeletal muscle and in macrophages associated with exacerbated inflammation (31), we speculated that altered fatty acid composition may contribute to the prolonged and exacerbated endometritis observed in *Srebf1^-/-^* mice. To address that possibility, uterine tissues were collected from WT or *Srebf1^-/-^* mice 7 days post LPS administration, after which total lipids were extracted and subjected to the quantitative analysis of fatty acids using liquid chromatography-tandem mass spectrometry (LC-MS/MS). Under the control (saline-treated) conditions, the abundances of major fatty acids, including C16:0 (palmitic acid), C18:0 (stearic acid), C16:1 (palmitoleic acid), C18:1 (oleic acid), C20:4 (arachidonic acid, AA), C20:5 (EPA) and C22:6 (DHA), were all reduced in the uterine tissues of *Srebf1^-/-^* as compared to WT mice (**Figure 2F**). In the WT mice, LPS treatment increased levels of several PUFAs, including C18:2 (linoleic acid), C20:4 (AA), C20:5 (EPA), and C22:6 (DHA), whereas it decreased levels of saturated and monounsaturated fatty acids, including C16:0, C18:0, C16:1 and C18:1 (**Figure 2F**). The shift toward PUFAs over saturated and monounsaturated fatty acids is consistent with previously observed changes in lipid metabolism during the resolution and reparative phases of inflammation after injury (17, 31, 32). In *Srebf1^-/-^* mice, those changes in the levels of fatty acids were greatly attenuated. In particular, the upregulation of PUFAs was not observed, and the levels of PUFAs remained lower than in WT tissues (**Figure 2F**).

### *Srebf1* deficiency increases intrauterine fetal death and resorbed embryo in CE

Given that CE is a strong risk factor for infertility, recurrent miscarriage and pregnancy complications in humans (8, 33), we next investigated the impact of endometritis and *Srebf1* deletion on fertility. Both male and female *Srebf1^-/-^* mice are fertile, and litter sizes (determined at 19.5-20.5 dpc (days post coitum)) were comparable between WT and *Srebf1^-/-^* mice when fed a fish meal-free diet (**Supplementary Figure 2**). The mean weights of the offspring were also similar between the genotypes (1.3 ± 0.1 g in WT, n = 5; 1.3 ± 0.1 g in *Srebf1^-/-^*, n = 6; p = 0.92), which is in agreement with an earlier report (34).

**Figure 3:**
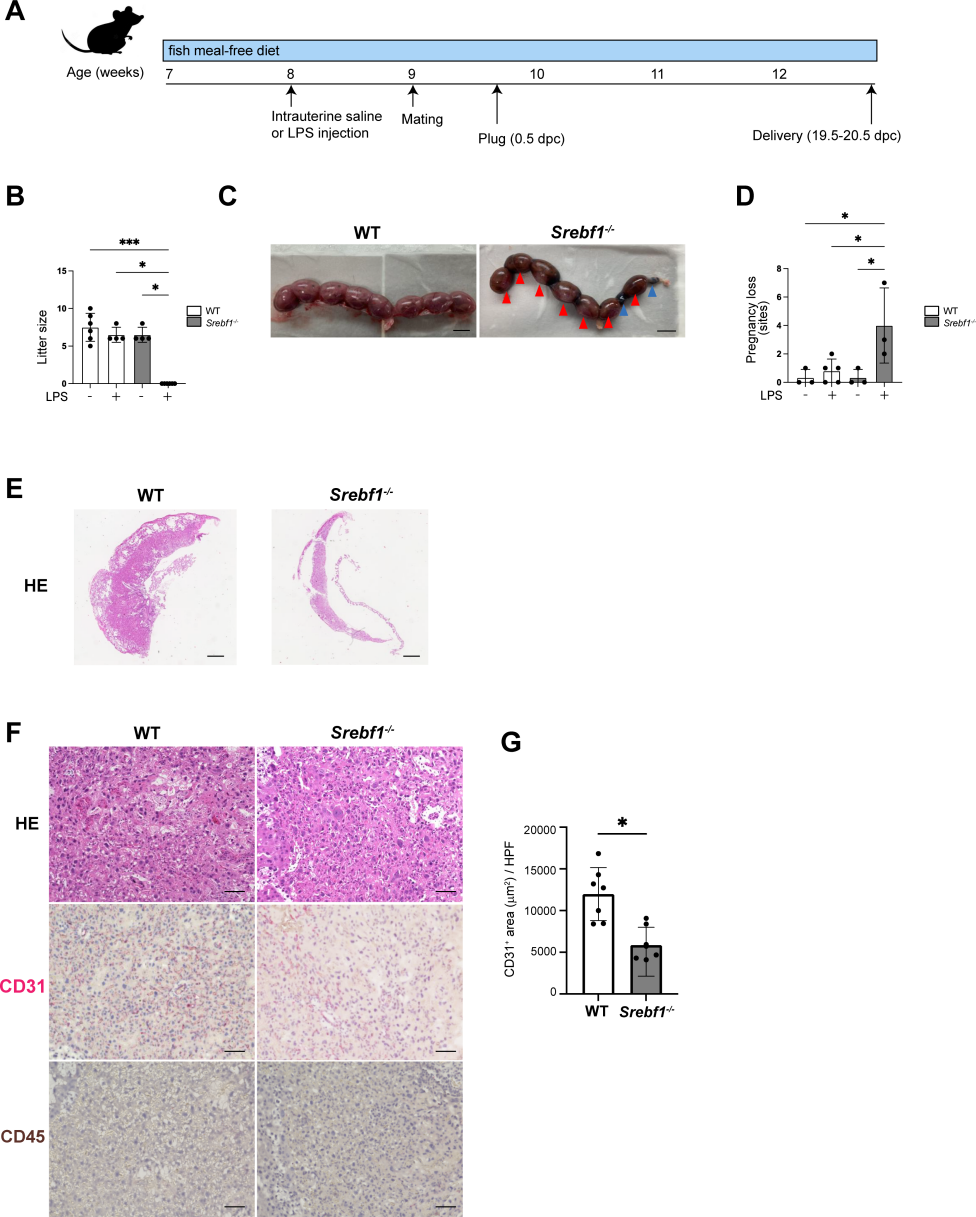
Systemic deletion of SREBP1 resulted in pregnancy loss after intrauterine LPS injection. **(A)** Experimental scheme. WT and *Srebf1^-/-^* female mice (7 weeks old) were fed a fish meal-free diet for 7 days, followed by intrauterine administration of LPS to induce endometrial inflammation. Seven days after LPS injection, the mice were mated with WT male mice. **(B)** Litter size (number of offspring) was evaluated 19.5-20.5 dpc. Data are presented as means ± SEM, and significance was determined using Kruskal-Wallis test followed by the Dunn's test. n = 4–6, *p < 0.05, ***p < 0.001. Only significant comparisons are shown. **(C)** Representative images of uteri collected from WT and *Srebf1^-/-^* mice 16.5 dpc. Blue arrowheads indicate resorbed embryos; red arrowheads indicate intrauterine fetal death. Scale bar: 10 mm. **(D)** Pregnancy loss (determined as the sum of the numbers of resorbed embryos and dead fetuses) was evaluated 16.5 dpc. Data are presented as mean ± SEM. and significance was determined using Kruskal-Wallis test, followed by the Dunn's test. n = 3-5, *p < 0.05. **(E, F)** Representative histological images of placentas harvested 16.5 dpc. Shown in E are low-magnification images of H-E-stained sections (scale bars, 200 μm); in F are high-magnification images of sections stained with H-E and immunostained for CD31 (red) and CD45 (brown) (scale bars, 50 μm). **(G)** CD31-positive areas in the labyrinth layers of placentas from female WT and *Srebf1^-/-^* mice 16.5 dpc. Data are presented as means ± SEM. Significance was determined using a Student’s *t*-test. *p < 0.05. n = 7 for each experimental condition.

To study the effect of endometrial inflammation on fertility, female *Srebf1^-/-^* and WT mice were administrated intrauterine LPS or saline 7 days prior to mating with WT male mice (**Figure 3A**). When mice were treated with saline, litter size and average offspring weight were comparable between the genotypes (**Figure 3B**). However, after LPS administration, whereas WT mice had average of 6.5 ± 0.9 offspring 19.5-20.5 dpc, *Srebf1^-/-^* mice had none (**Figure 3B**). At 16.5 dpc, the uteri of WT mice contained healthy offspring with a bright red appearance. In sharp contrast, the uteri of *Srebf1^-/-^* mice appeared dark red with accompanying intrauterine fetal death and hematoma formation (red arrowhead; **Figure 3C**). We also evaluated pregnancy loss by examining the uteri at 16.5 dpc (**Figure 3D**). Pregnancy loss was defined as the sum of miscarriages during early pregnancy, indicated by a resorbed embryo (blue arrowhead), and intrauterine fetal death during late pregnancy (red arrowhead; **Figure 3C**) (35, 36). In the saline-administered group, the prevalence of pregnancy loss was comparable between the two genotypes. However, in the LPS-administered group, although successful implantation was observed, the prevalence of pregnancy loss was significantly higher in *Srebf1^-/-^* than WT mice (**Figure 3D**).

Placental hypoplasia and impaired vascularization causes intrauterine fetal death in both humans and mice. Histologically, the miscarried placenta of *Srebf1^-/-^* mice displayed thinning of the placental parenchyma at 16.5 dpc, indicating significant placental hypoplasia (**Figure 3E**). Although no accumulation of CD45^+^ mononuclear cells was evident, immunostaining for CD31 revealed a significant reduction in the CD31^+^ capillary area in placentas from *Srebf1^-/-^* mice as compared to WT mice (**Figures 3F, G**). These findings suggest that placental hypoplasia with deficient vascularization contributed to the increased intrauterine fetal death during late pregnancy, which in turn contributed to the pregnancy loss in the mice.

### EPA ameliorates inflammation and miscarriage in *Srebf1^-/-^* mice

**Figure 4:**
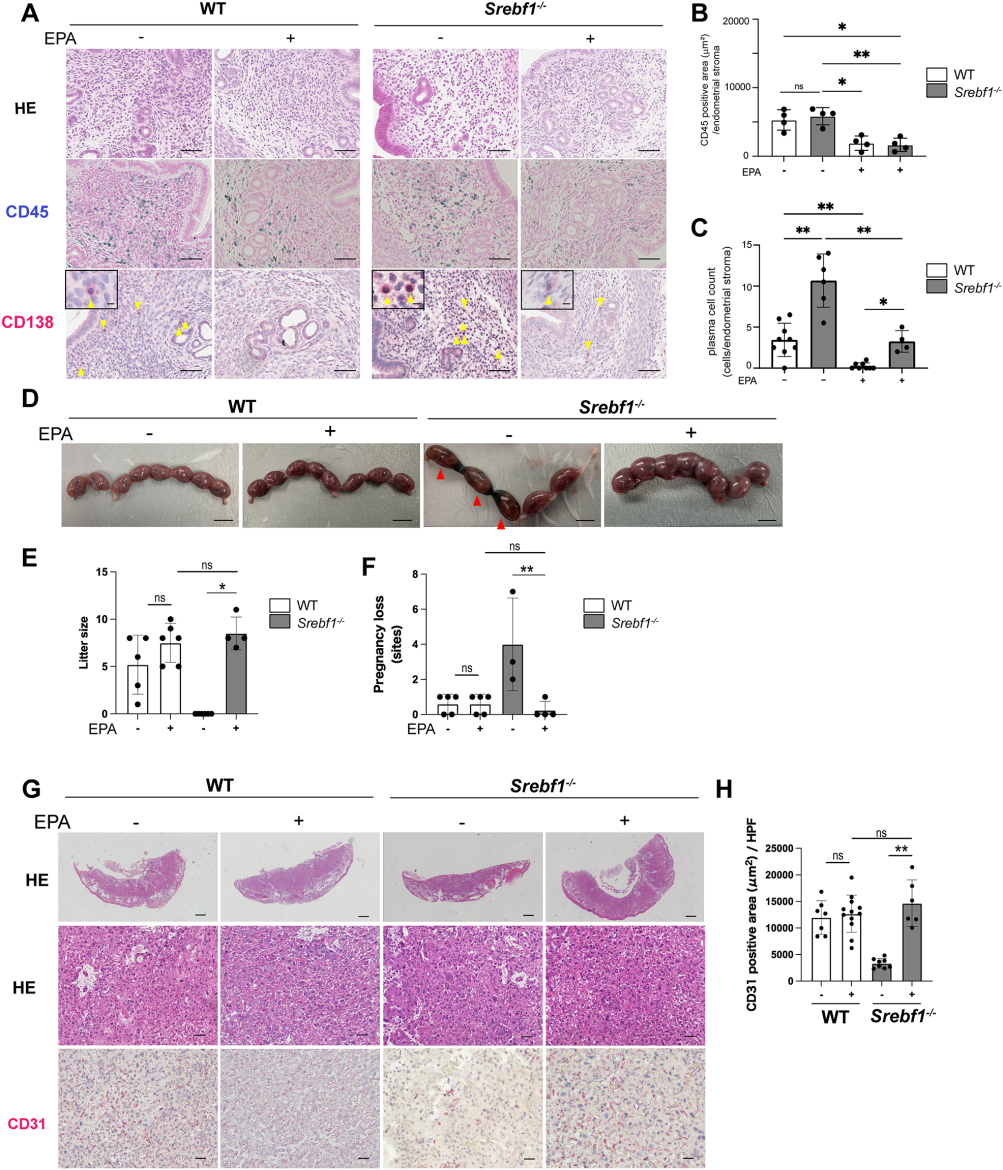
EPA supplementation ameliorates chronic endometritis, pregnancy loss and placenta-associated obstetric complications in *Srebf1^-/-^* mice. **(A)** Representative histological images of the endometrial stromal area of uteri collected from WT and *Srebf1^-/-^* mice 7 days after saline or LPS injection (scale bars: 50 µm). Yellow arrowheads on sections immunostained for CD138 show plasma cells. Magnified images are in the upper left (scale bars: 10 µm). **(B, C)** CD45-positive areas **(C)** and plasma cell counts **(D)** in the endometrial stromal area of uteri collected from WT and *Srebf1^-/-^* mice on day 7 after LPS administration. Mice were fed a fish meal-free diet (control) or 5% EPA diet. Data are presented as means ± SEM, and significance was determined using Kruskal-Wallis test followed by the Dunn's test. *p<0.05, **p<0.01, ns; not significant. n = 4–5 for each experimental condition. Two sections were made from each side of the bicornuate uterus per mouse. **(D)** Representative images of uteri collected 16.5 dpc from WT and *Srebf1^-/-^* mice fed with 5% EPA diet or control (fish meal-free) diet. Red arrowheads show intrauterine fetal death (scale bar: 10 mm). **(E)** Numbers of offspring (litter size) evaluated 19.5-20.5 dpc. Data for litter size without EPA supplementation are identical as those shown in **Figure 3**. Data are presented as means ± SEM. Significance was determined using Kruskal-Wallis test followed by the Dunn's test. n = 4-6, *p < 0.05. **(F)** Pregnancy loss 16.5 dpc. Data are presented as means ± SEM. Significance was determined using Kruskal-Wallis test followed by the Dunn's test. n = 3-5, **p < 0.01. **(G)** Representative histologic images of placenta stained with H-E and immunostained for CD31 (scale bars: 200 μm for upper panels, 50 μm for middle and lower panels). **(H)** CD31-positive area within the labyrinth layers of placentas collected from WT and *Srebf1^-/-^* mice 16.5 dpc. Data are presented as means ± SEM. Significance was determined using Kruskal-Wallis test followed by the Dunn's test. **p < 0.01. n = 6–8 for each experimental condition.

Given that *Srebf1* deficiency reduces PUFAs in endometritis tissues and that abnormal PUFA metabolism may contribute to the observed inflammation and placental abnormality and pregnancy loss (**Figures 2**, **3**), we investigated the effects of dietary EPA supplementation. Mice were fed either a control (fish meal-free) or a 5% EPA-enriched diet for 7 days, followed by the intrauterine LPS injection. Uterine tissue was then collected on day 7 post injection and subjected to histological analysis. H-E staining revealed no significant differences between the two groups. However, feeding of the EPA-rich diet led to reduced accumulation of CD45^+^ mononuclear cells and CD138^+^ plasma cells in the subepithelial stromal regions of endometria from both WT and *Srebf1^-/-^* mice (**Figures 4A–C**). Notably, CD138^+^ cell counts in *Srebf1^-/-^* mice on the EPA diet were decreased to levels comparable to those in WT mice on the control diet (**Figures 4A**–**4C**). Moreover, litter sizes were increased and pregnancy losses were reduced in *Srebf1^-/-^* mice fed the EPA-rich diet (**Figures 4D–F**). EPA supplementation also prevented the placental thinning induced by LPS and increased CD31^+^ capillary area (**Figures 4G, H**). Litter size and the number of pregnancy losses were not changed by EPA administration, and the vascular area of the placenta determined by the CD31 immunostaining was not significantly increased by EPA administration, suggesting that the beneficial effects of EPA supplementation on pregnancy outcome and placenta were not evident in the WT mice (**Figures 4D-H**). Taken together, these findings indicate an EPA-rich diet ameliorates the sustained intrauterine inflammation and increases litter size in *Srebf1^-/-^* mice.

### EPA diet modulates the phospholipid composition of uterine endometrium

**Figure 5:**
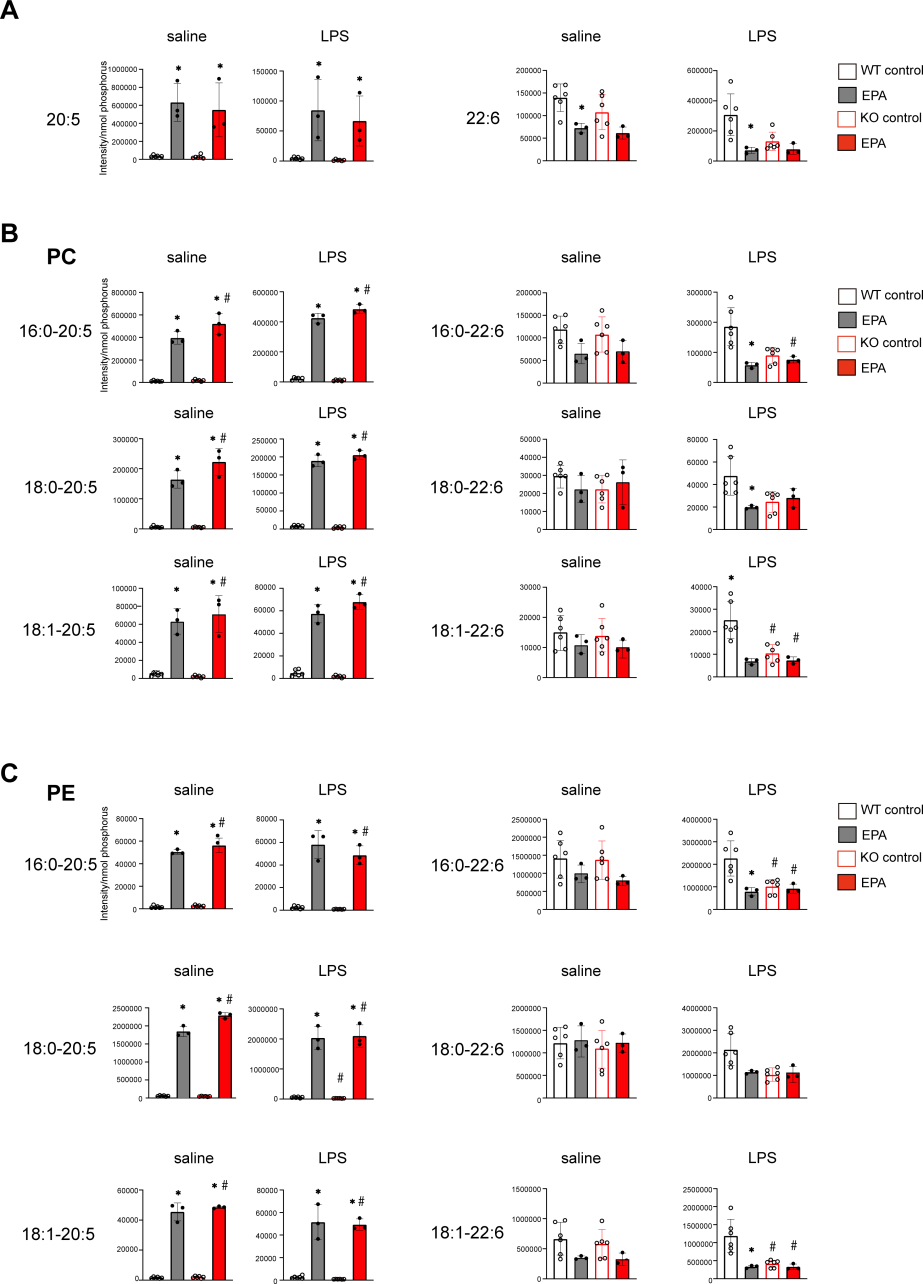
Phospholipid remodeling caused by exogenous EPA supplementation. **(A)** Quantitative analysis of omega-3 free fatty acids in uterine tissues collected 7 days after intrauterine administration of saline or LPS following 5% EPA supplementation. Levels of EPA (C20:5) and DHA (C22:6) are shown. Data are presented as means ± SEM. Significance was determined using one-way ANOVA with Tukey’s *post-hoc* test; *p<0.05. n=3-6/experimental condition. **(B, C)** Phosphatidylcholine (PC, in B) and phosphatidylethanolamine (PE, in C) containing EPA (C20:5) and DHA (C22:6) as sn-2 fatty acids were analyzed with LC-MS. Data are shown as means ± SEM and were analyzed using one-way ANOVA with Tukey’s *post-hoc* test in all panels where P values are shown. n =3–6 for each group. *P <0.05 vs. the same genotype fed a control diet, ^#^P <0.05 vs. WT fed a control diet. ns: not significant.

Because we previously showed that diet supplementation with EPA alters the composition of phospholipids in macrophages (31), we assessed the levels of EPA (C20:5) and DHA (C22:6) in uterine tissues from WT and *Srebp1^-/-^* mice on day 7 following LPS treatment. The EPA-rich diet increased free EPA levels, irrespective of genotype or treatment. On the contrary, DHA levels were decreased by the EPA-rich diet in WT mice and tended to be decreased in *Srebp1^-/-^* mice (**Figure 5A**). We then analyzed the fatty acid composition of phospholipids, focusing on phosphatidylcholine (PC) and phosphatidylethanolamine (PE), which are the major phospholipid species in mouse endometrium (37). We found that EPA supplementation significantly increased the amounts of PC and PE species containing EPA (C20:5) at the *sn*-2 position in both WT and *Srebf1^-/-^* mice (**Figures 5B, C**), which suggests phospholipid remodeling. On the other hand, the levels of PC and PE containing DHA (C22:6) tended to be decreased or unchanged (**Figures 5B, C**).

### Phospholipids containing EPA are decreased in endometrium tissue from patients with CE

**Figure 6:**
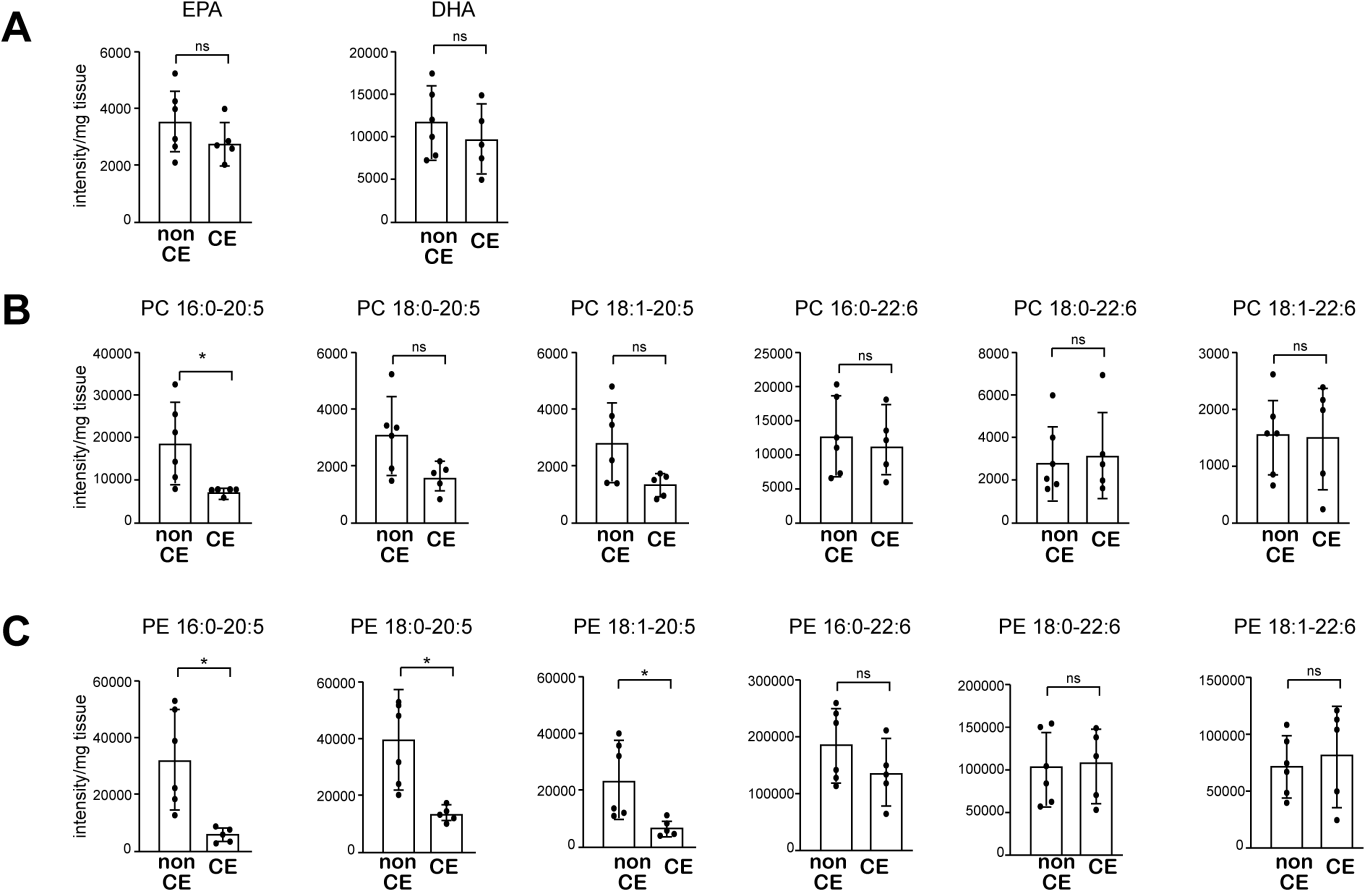
The levels of major EPA-containing phospholipids are decreased in endometrial tissue from patients with CE. **(A)** Levels of free EPA and DHA were analyzed using LC-MS. **(B, C)** Levels of PC **(B)** and PE **(C)** containing 20:5 (EPA) and 22:6 (DHA) were analyzed using LC-MS. Data are presented as means ± SEM, and statistical significance was determined using Student’s *t*-tests. *p < 0.05, ns, not significant. non CE: n=6, CE: n=5.).

Finally, we asked whether altered uterine phospholipid content may also be associated with the pathogenesis of CE in humans. To address that question, we performed lipidomic analysis of endometrial biopsy samples from 11 women who experienced either recurrent implantation failure (RIF) or recurrent pregnancy loss (PRL). The endometrial biopsy was performed during the implantation period, 5–7 days after ovulation. Among those patients, 5 met the diagnostic criteria for CE (i.e., detection of CD138^+^ cells in the stromal region of an endometrial sample obtained through biopsy), while the other 6 patients did not (**Supplementary Table**). All of the patients were of Asian descent, with a mean age of 37.2 years, and none had a history of childbirth or previous antibiotic treatment for CE. While the total free EPA levels did not differ between CE and non-CE patients (**Figure 6A**), the levels of major EPA-containing phospholipid species, including PC 16:0-20:5, and PE 16:0-20:5, 18:0-20:5, and 18:1-20:5, were significantly lower in CE patients compared to non-CE patients (**Figures 6B, C**). In contrast, both free DHA levels as well as DHA-containing phospholipids (PC and PE) did not differ significantly between the two groups (**Figures 6A–C**). Thus, lower endometrial levels of EPA-containing phospholipid species were associated with CE in humans, as we observed in the mouse model of CE.

## Discussion

This study highlights the role of dysregulated lipid metabolism in CE and its impact on pregnancy outcomes. *Srebf1* ^-/-^ mice exhibited altered endometrial fatty acid composition, especially reduction in EPA-containing phospholipids, and showed sustained endometrial inflammation and CD138^+^ plasma cell infiltration, mimicking CE. *Srebf1* ^-/-^ mice also showed pregnancy loss with placental abnormality without affecting implantation in the endometritis model. Dietary EPA supplementation increased the levels of endometrial EPA- and EPA-containing phospholipids, suppressed inflammation and pregnancy loss, and improved placental function in *Srebf1* ^-/-^ endometritis, which suggests that dysregulation of PUFA (EPA in particular) metabolism due to *Srebf1* deficiency contributes to prolonged endometrial inflammation and miscarriage. Finally, levels of EPA-containing phospholipids were decreased in endometrial tissue from CE patients, suggesting the involvement of impaired PUFA metabolism in the pathogenesis of CE in humans.

CE is diagnosed based on the presence (or accumulation) of plasma cells within the endometrial stroma due to unknown causes. Intrauterine injection of LPS induced acute leukocyte accumulation on day 1 after administration, which was followed by CD138^+^ plasma cell accumulation within the endometrial stroma (**Figure 1**). In WT mice, CD138^+^ cell accumulation peaked on day 3, and the lipid content shifted toward PUFAs, which marks the resolution phase of inflammation. This suggests the acute inflammation started to resolve after day 3 in WT mice. By contrast, *Srebf1* deletion led to sustained plasma cell accumulation, even on day 7, enhanced inflammatory gene expression, and suppression of the shift in the endometrial fatty acid composition toward PUFAs. This indicates that, even as late as day 7, the inflammation remained unresolved in *Srebf1* ^-/-^ mice. Importantly, LPS-induced inflammation in *Srebf1* ^-/-^ mice also induced pregnancy loss and placental abnormality. These findings demonstrate that intrauterine administration of LPS to *Srebf1* ^-/-^ mice recapitulated key features of human CE. Although a model of endometritis induced by LPS injected into the uterus of mice has been reported, it did not recapitulate the pathology of chronic endometritis in humans (38, 39). Our CE mouse model has the potential to accelerate the study of the molecular mechanism of CE and to facilitate the development of novel therapeutic interventions.

The mechanism that regulates CD138^+^ plasma cell accumulation and their functional roles in CE remain poorly understood. However, various observational studies on clinical outcomes have suggested their pathological roles in CE pathology. Several studies indicated that lower live birth and pregnancy rates were observed in patients diagnosed with CE by the accumulation of CD138^+^ cells in their endometrium (5, 40), although one study reported no significant difference in pregnancy rates between CD138-positive and CD138-negative cases (41). Furthermore, high CD138^+^ cell counts are positively correlated with poorer reproductive outcomes including lower live birth and pregnancy rates (5, 40, 42). Additionally, CD138-positive cell infiltration has been shown to associate with endometrial polyps and alterations in molecular markers such as TGF-β1, MMP-9, and αvβ3 integrin, which are associated with endometrial dysfunction and prolonged inflammation (40).

Our research indicates that chronic endometritis in SREBP1 deficiency leads to the pregnancy loss accompanied by the placental abnormality (**Figure 3**). Previous studies have suggested that arachidonic acid metabolites play a crucial role in female reproduction. For instance, deficiencies of molecules involved in arachidonic acid metabolism and its signaling pathway, such as cytosolic phospholipase A2 (43), cyclooxygenase-2 (44) and prostaglandin E_2_ receptor (45) resulted in female infertility. In our study, uterine tissue from *Srebf1^-/-^* mice showed a reduction in arachidonic acid (C20:4) levels (**Figure 2F**), which may explain, at least in part, pregnancy loss (fetal death) associated with *Srebf1* deficiency. Furthermore, we found that the adverse effects of SREBP1 deficiency in the context of chronic endometritis could be ameliorated by EPA supplementation. This suggests that the beneficial effects of EPA may be partly due to its ability to inhibit persistent inflammation. Multiple pathways have been reported to link inflammation to implantation and placental impairment (46). These include dysregulation of cytokines, altered composition of immune cells required for the physiological processes of pregnancy, and altered endometrial cell differentiation, proliferation and apoptosis. It is therefore essential to identify the specific processes that EPA modulates to suppress miscarriage. In addition, the increase in phospholipid species containing EPA suggests EPA supplementation triggers phospholipid remodeling, which may affect the function of not only immune cells, but also endometrial epithelial cells and stromal cells potentially contributing to the restoration of decidualization processes. In that regard, EPA reportedly ameliorates pulmonary endothelial dysfunction in particulate matter air pollution-induced inflammation, in part by improving eNOS coupling (47). In future experiments, it will be important to assess the effects of EPA on the various cell types comprising the endometrium and their interactions.

Recent studies have suggested that abnormalities in fatty acid metabolism may impair endometrial receptivity and contribute to adverse pregnancy outcomes (48). In particular, an imbalance in the n-3/n-6 PUFA ratio promotes excessive prostaglandin production and induce a pro-inflammatory endometrial environment that is prevent implantation (49). In the present study, although implantation efficiency was not directly assessed, EPA may have improved not only fetal survival but also endometrial receptivity and implantation efficiency, thereby contributing to the increased number of offspring. This interpretation is suggested by the observation that the litter size in the EPA-treated group of the SREBP1-deficient CE mouse model exceeded the number expected from the prevention of fetal loss alone, and that EPA treatment also showed a trend toward increased litter size even in wild-type mice, which rarely exhibit pregnancy loss (**Figures 4E, F**). These observations support the possibility that abnormal lipid metabolism in chronic endometritis negatively affects not only pregnancy maintenance but also implantation. Future studies are needed to assess the effects of EPA on and the roles of lipid metabolism in implantation.

We found that endometrial tissue from patients diagnosed with CE had decreased levels of EPA-containing phospholipids, although there was no significant difference in the amount of free EPA (**Figure 6**). Less than 5% of fatty acids exist as free fatty acids in the body, and about 30% of fatty acids exist as phospholipids (LIPID MAPS Structure Database. (https://www.lipidmaps.org)). Accordingly, our results support the notion the dysregulation of PUFA metabolism is associated with CE. In mice, orally ingested EPA is incorporated into the phospholipid membranes of cells constituting the endometrium, increasing the EPA-containing phospholipids in the endometrium (**Figures 6B, C**). It is expected that consumption of an EPA-rich diet increases the EPA-containing phospholipids in the endometrium in humans, as previous studies have shown that oral EPA increases plasma and tissue level of EPA-containing phospholipids (50, 51). The results of the present study suggest that oral EPA ameliorate CE development that involves dysregulated lipid metabolism. Future studies need to further analyze dysregulation in lipid metabolism in CE and other conditions associated with poor pregnancy outcomes.

Several human studies have evaluated the effects of PUFAs, including EPA and DHA, on pregnancy rate (52), pregnancy outcome (53), gestational diabetes, preeclampsia (54), fetal growth and prolonged gestational age as well as the weight, height and head circumference of infants (55). However, a causal relationship between PUFAs and pregnancy outcomes remains unclear. To our knowledge, no studies have investigated the relationship between PUFA intake and pregnancy outcomes in patients with CE. Our mouse model of CE and the present findings that point to the involvement of SREBP1-mediated fatty acid metabolism in CE and pregnancy loss could facilitate the elucidation of the molecular mechanisms underlying CE and the actions of dietary PUFAs in pregnancy.

A limitation of this study is the small sample size of lipidomic analysis using endometrial samples from CE patients, which included individuals diagnosed with either RIF or RPL. Our CE mouse model primarily exhibited fetal loss rather than implantation failure. Therefore, analyzing endometrial samples specifically from CE patients with PRL would better align with our mouse model when assessing the role of fatty acid metabolism in chronic endometritis. In future studies, large-scale lipid analyses with clinically well-characterized cohorts will be necessary to further elucidate how dysregulated lipid metabolism contributes to different types of adverse pregnancy outcomes associated with CE. In conclusion, the results of the present study strongly suggest that dysregulated fatty acid metabolism, particularly SREBP1-mediated pathways, drives chronic endometrial inflammation and adverse pregnancy outcomes. These findings underscore the therapeutic potential of targeting fatty acid metabolism, particularly through EPA supplementation, to prevent post-implantation fetal loss and miscarriage associated with CE. These insights have important implications for understanding the pathophysiology of CE and may pave the way for novel therapeutic strategies for the management of CE and related pregnancy complications.

## Materials and methods

### Animals

All mice used in this study had a C57BL/6 background. *Srebf1^-/-^* mice were generated as described previously and provided by Dr. Hitoshi Shimano (The University of Tsukuba) (34). All mice were maintained in an institutional animal facility with a 12 h/12 h light-dark cycle and free access to food and water. All experimental procedures were performed following the research guidelines for the care and use of laboratory animals at the Nippon Medical School.

### Establishment of a chronic endometritis model in mice

Seven-week-old female mice were fed fish meal-free diet (fish meal-free MF: 4.4% fat; Oriental Yeast Co., Ltd., Tokyo, Japan), as described previously (26). For studies involving supplemental EPA supplementation, mice were fed a fish meal-free diet supplemented with 5% EPA ethyl ester (v/v) (9% EPA; Bizen Chemical Co., Ltd. Okayama, Japan) for 7 days before intrauterine LPS administration. LPS or saline (as a control) was administered to the uteri of 8-week-old female mice following the technique used for intrauterine implantation of blastocysts (56). Briefly, the abdomen was opened under general anesthesia, and 10 µg of LPS (Sigma) dissolved in 100 µL of saline were administered from each uterine horn into the uterus through a 29G needle, followed by closure of the abdomen. In the control group, 100 µL of saline were administered to the uterus. After euthanasia by cervical dislocation on days 1, 3, 5 and 7 after LPS or saline administration, the lower abdomen was incised, and the uterus was removed and observed. We injected 10 µg of LPS per uterine horn (20 µg per mouse) to induce chronic endometritis, because previous studies demonstrated that the administration of 20–40 µg of LPS induced acute endometritis (38), while 20 µg of LPS induced inflammatory preterm birth in pregnant mice (57). We confirmed that 20 µg LPS successfully induced chronic inflammation without systemic toxicity such as significant weight loss.

For studies to evaluate pregnancy loss, male WT mice aged ≥ 8 weeks were housed with the female mice daily beginning 7 days after LPS or saline administration. The day a vaginal plug was observed in the morning was identified as 0.5 dpc, and the uterus was collected at 16.5 dpc. After euthanasia by cervical dislocation, the lower abdomen was incised, and the pregnant uterus was removed and observed.

### Preparation and immunostaining of uterine tissue sections

Whole mouse uterus were fixed by immersion in Tissue-Tek Ufix (Sakura Finetek, Tokyo, Japan), embedded in paraffin blocks, and cut into 10-µm-thick cross-sectional slices. The sections were then deparaffinized, rehydrated, stained with hematoxylin for 10 min, washed with running water for 15 min, stained with eosin for 5 min, and quickly washed in a 70, 80, 90, 95 and 100% ethanol series before washing twice in xylene.

To obtain immunofluorescence images, tissue sections were immersed in Tris-EDTA buffer (pH 9.0) solution heated to 95 °C and boiled for 20 min. After washing three times in phosphate-buffered saline (PBS; 5 min), the sections were blocked with 2% BSA for 2 h at room temperature. The samples were then reacted with primary antibodies (anti-CD138/Syndecan-1 antibody (#10593-1-AP, Proteintech), CD45 (#550539, BD) and CD31 (N1596, DAKO) at 4°C overnight and again washed three times with PBS (5 min). Thereafter, the sections were incubated with secondary antibody for 30 min at room temperature and washed with PBS (3x, 5 min) before being incubated with an alkaline phosphatase kit (AK-5000, Vector Laboratories, US) for 30 min at room temperature. After again washing with PBS (3x, 5 min), the chromogenic reaction was carried out for 30 min using Vector Red (#SK-5105, Vector Laboratories, US) as a substrate. The sections were then cleared with xylene and mounted.

Images of immunofluorescence were acquired using a microscope (BZ-X810, Keyence, Osaka, Japan) and analyzed. The numbers of CD138^+^ cells present in the entire interstitial region surrounding the uterine lumen were counted, and the CD45^+^ and CD31^+^ areas are measured. Four sections from the bicornuate uterus of each mouse were made and analyzed.

### Lipidomic analysis

MS-based lipidomic analysis was performed using our published protocol (58). Briefly, for phospholipid detection, frozen uterine tissues were crushed in a Multi-Beads-Shocker™ (Yasui Kikai, Osaka, Japan) at 2,500 rpm for 15 s (2 cycles), with a pause time of 5 s. Lipids were extracted from the crushed tissue using the Bligh and Dyer method (59). Electrospray ionization (ESI)-MS analysis was performed using a 4000Q-TRAP, a triple quadrupole-linear ion trap hybrid mass spectrometer (Sciex, Framingham, MA, USA) equipped with reverse-phase LC (NexeraX2 system, Shimadzu, Kyoto, Japan). Samples were injected using an autosampler connected to a Kinetex C18 column (2.1 × 150 mm, 1.7 µm particle, Phenomenex, Torrance, CA, USA) coupled to the ESI-MS and separated using a step gradient of mobile phase A (acetonitrile/methanol/water = 1:1:1 [v/v/v] containing 5 µM phosphoric acid and 1 mM ammonium formate) and mobile phase B (2-propanol containing 5 µM phosphoric acid and 1 mM ammonium formate) at a flow rate of 0.2 mL/min at 50 °C.

Lipids were identified based on multiple reaction monitoring (MRM) transitions and retention times. Quantification was performed based on the peak area of the MRM transition and a calibration curve constructed using an authentic standard for each compound. As internal standards, d5-labeled EPA (50 pmol), LPC17:0 (25 pmol) and PE14:0-14:0 (25 pmol) (Cayman Chemical, Ann Arbor, MI, USA) were added to each sample.

### RNA-seq

Three biological replicates (n=3) were used for RNA-seq analyses for each group: Saline, LPS, EPA, and EPA+LPS, in WT and *Srebf1^-/-^* mice. Poly-A mRNA was extracted from total RNA using a NEBNext poly(A) mRNA magnetic isolation module (New England Biolab), and RNA-seq libraries were prepared using a NEBNext Ultra RNA Library Prep kit for Illumina according to the manufacturer’s protocol (New England Biolab). The libraries were then PCR-amplified for approximately 12 cycles and sequenced on a Novaseq (Illumina). Reads were aligned to the mm10 mouse genome using STAR (60). Expression analysis of the RNA-seq data was performed using HOMER (61). GSEA (30) was performed using rank files generated from expression data analyzed using DESeq2 (62). All RNA-seq data are available in the GEO under accession number GSE278134.

### Human endometrial tissue collection

Endometrial tissue was collected from 11 patients with a history of recurrent implantation failure (RIF, n=5) or recurrent pregnancy loss (RP, n=6) who underwent endometrial biopsy. Endometrial biopsy was performed during the implantation period, 5–7 days after ovulation at the Department of Obstetrics and Gynecology, Nippon Medical School Hospital between 2021 and 2023. The study protocol was approved by the institutional review board of Nippon Medical School Hospital (No. B-2021-393). All patients provided written informed consent for the use of their endometrial samples in this research.

### Statistical analysis

Data are presented as means ± standard error of the mean (SEM). Statistical significance was determined using two-tailed Student’s *t*-tests. Two-way analysis of variance (ANOVA) with *post-hoc* Bonferroni’s multiple comparison test or the Kruskal-Wallis test followed by Dunn’s test was used in experiments involving multiple factors. p < 0.05 was considered statistically significant. All statistical analyses were performed using Prism 9 software (GraphPad, San Diego, CA, USA).

## Data Availability

All RNA-seq data are available in the GEO under accession number GSE278134.
