# Supplementary material for: Impaired SREBP1-mediated regulation of lipid metabolism promotes inflammation in chronic endometritis
Source: Front Immunol. 2025 Jun 5;16:1547949. doi: 10.3389/fimmu.2025.1547949 (PMC12176762; doi:10.3389/fimmu.2025.1547949)
Supplement: Supplementary file 1 [file DataSheet1.pdf]

Supporting Information

Supplemental Table

Profile of patients who underwent endometrial biopsy

Indication for endometrial biopsy	
RIF, n	5
PRL, n	6
BMI, kg/m^2	21.9 (16.5-32.0)
Past pregnancy, times	1.9 (0-4)
Diagnosis of chronic endometritis	
Positive, n	5
Negative, n	6

Supplemental Figure legends

Supplemental Figure 1

Representative images showing H-E staining and immunostaining for CD138 in the subepithelial stromal region of the endometrium in C57BL/6 mice (scale bars, 100 μm).

Supplemental Figure 2

Litter sizes in *Srebf1*$^{-/-}$ and control mice fed a regular diet

Litter sizes were determined 19.5-20.5 dpc. Data are presented as means ± SEM, and significance was determined using Student's *t*-test. ns, not significant.

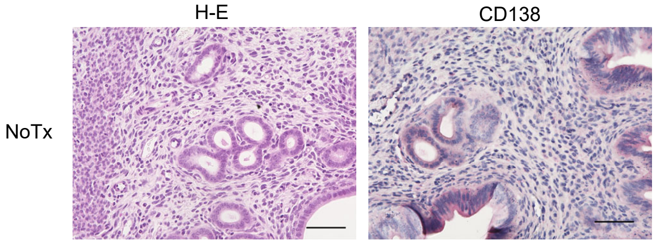

Suppl. Fig. 1

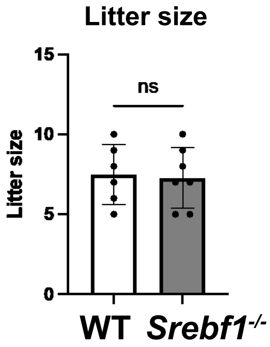

Suppl. Fig. 2
